# Three-Dimensional Titanium Substrates with Anodic TiO_2_ Layers for Enhanced Time-Dependent Corrosion Protection in Biomedical Environments

**DOI:** 10.3390/molecules31172959

**Published:** 2026-08-24

**Authors:** Małgorzata Fus, Jakub Skibiński, Agnieszka Chmielewska-Wysocka, Wojciech Święszkowski, Grzegorz Dariusz Sulka, Magdalena Jarosz

**Affiliations:** 1 Department of Physical Chemistry & Electrochemistry, Faculty of Chemistry, Jagiellonian University, Gronostajowa 2, 30-387 Krakow, Poland; 2 Doctoral School of Exact and Natural Sciences, Jagiellonian University, Lojasiewicza 11, 30-387 Krakow, Poland; 3 Institute of Heat Engineering, Faculty of Power and Aeronautical Engineering, Warsaw University of Technology, Nowowiejska 21/25, 00-665 Warsaw, Poland; 4 Multidisciplinary Research Center, Cardinal Stefan Wyszynski University in Warsaw, Marii Konopnickiej 1, 05-092 Dziekanow Lesny, Poland; 5 Materials Design Division, Faculty of Materials Science and Engineering, Warsaw University of Technology, Woloska 141, 02-507 Warsaw, Poland; 6 Independent Researcher, Kazimierza Wielkiego 140, 30-074 Krakow, Poland

**Keywords:** nanostructured titanium dioxide, anodization, corrosion resistance, electrochemical impedance spectroscopy, bioimplants

## Abstract

Enhancing the performance of titanium biomaterials remains a critical challenge in the development of durable implant materials, particularly under complex physiological conditions where corrosion processes are influenced by interactions with biological species. Electrochemical oxidation has emerged as a promising approach for generating nanostructured titanium dioxide layers, which can improve corrosion resistance. In this study, nanostructured oxide layers were synthesized on additively manufactured 3D titanium scaffolds via anodization in a fluoride-containing ethylene glycol and water electrolyte. Corrosion resistance was systematically evaluated using open-circuit potential measurements, Tafel analysis, and electrochemical impedance spectroscopy, considering the effects of biological medium composition and prolonged exposure to corrosive conditions. The main scientific contribution of this work is the elucidation of the time-dependent corrosion behavior and electrochemical stability of anodized additively manufactured titanium scaffolds under physiological exposure conditions. The results demonstrated that the medium composition significantly influenced the properties of the anodized materials, primarily due to the adsorption of medium species on the nanostructured surface. Prolonged exposure tests further confirmed the superior durability of the coatings, which is attributed to the formation of a protective protein layer that enhances corrosion resistance in aggressive environments. These findings advance the understanding of time-dependent corrosion behavior in complex biological environments and highlight the effectiveness of nanostructured oxide layers in maintaining the electrochemical stability of titanium biomaterials during prolonged exposure. Combined with additive manufacturing, this approach represents a promising route toward the development of patient-specific implants with enhanced durability and long-term functionality for bone regeneration applications.

## 1. Introduction

Metals commonly used in the production of prosthetic materials include titanium, titanium alloys, stainless steel, and Co-Cr-Mo alloys [1,2]. Among these, titanium is particularly valued in orthopedics due to its favorable combination of mechanical strength, high corrosion resistance, and excellent biocompatibility, which supports successful osseointegration [3]. Nevertheless, one major limitation of pure titanium is its relatively high elastic modulus (approximately 120 GPa [4]), which is significantly higher than that of natural bone, typically ranging from 10 to 30 GPa [5]. This mechanical mismatch can cause stress shielding, leading to localized stress concentrations and ultimately reducing the service life of the implanted prosthesis [6,7]. One strategy to overcome this stiffness mismatch is the use of titanium alloys, among which Ti-6Al-4V is the most widely applied [8,9,10]. However, its use has been associated with adverse effects such as neuropathy and Alzheimer’s disease, linked to the release of aluminum and vanadium ions, the latter being particularly toxic [11,12]. As a result, research has increasingly focused on alternative titanium alloys as well as pure titanium modified through morphological design. A proven method of reducing the stiffness of metallic titanium is to increase its porosity. In this context, additive manufacturing (AM) has gained prominence, as it allows precise control over structural parameters such as pore size and distribution, enabling the mechanical performance to be tailored to closely match that of bone. Moreover, AM ensures full control of the fabrication process, allowing the production of patient-specific implants designed for individual treatment needs, which can enhance their clinical effectiveness. Beyond mechanical compatibility, increased porosity also mimics the natural structure of bone, facilitating faster osseointegration and offering additional functionalities, such as serving as a platform for localized drug delivery [13,14].

Importantly, the combined effects of wear and corrosion significantly compromise the durability of metallic implants in the human body, often necessitating revision surgeries [15]. Corrosion not only compromises the structural integrity of prostheses but also alters the surrounding biological environment. The release of metal ions may further trigger allergic reactions and cause systemic toxicity [16]. Therefore, investigating corrosion behavior is essential for the development of durable and biocompatible metallic biomaterials.

The inherent high corrosion resistance of titanium is attributed to the spontaneous formation of a dense and stable oxide film, primarily TiO_2_, when exposed to air or oxygen-containing environments [17,18]. However, this oxide layer often contains defects, driving ongoing research toward methods that produce more uniform and compact protective coatings. Anodization, an electrochemical technique, is one of the most effective strategies to generate a nanostructured, compact oxide layer on titanium [19]. Compared with other TiO_2_ deposition techniques, such as sol–gel processing, physical vapor deposition (PVD), and chemical vapor deposition (CVD), anodization enables the direct electrochemical growth of an oxide layer from the titanium substrate. It also offers convenient control over the morphology and dimensions of the resulting oxide layer by adjusting parameters such as the applied potential, anodization time, and electrolyte composition [20]. These features make anodization particularly attractive for the surface modification of titanium substrates with complex geometries, where direct oxide formation and precise control over surface morphology are advantageous. Moreover, this method allows a marked increase in the surface area of the implant, thereby potentially enhancing the material’s bioactivity and promoting favorable interactions with biological systems. Such nanostructured oxide layers also provide versatile platforms for further functionalization. For instance, Ma et al. demonstrated that incorporating a carbon monoxide-releasing molecule (CORM-401) into anodically formed TiO_2_ nanotubes enhanced surface hydrophilicity and albumin-selective adsorption [21], resulting in reduced platelet adhesion, lower hemolysis, and improved hemocompatibility of cardiovascular implants.

Extensive studies have examined the corrosion resistance of titanium and its alloys, particularly Ti-6Al-4V, and the role of surface oxide layers in improving performance [22,23,24]. For instance, Banea et al. reported that electrochemical oxidation significantly improved the anticorrosive properties of Ti-6Al-4V under inflammatory conditions [25]. By contrast, far fewer studies have investigated the anticorrosive behavior of oxide layers formed on commercially pure titanium. Otadi et al. combined channel angular pressing with a two-step anodization process to produce titanium oxide nanotube coatings, showing that the resulting nanostructured layers suppressed charge transfer, prevented pitting corrosion, and provided superior protection with increasing thickness [26]. However, their work mainly focused on optimizing synthesis parameters, with limited evaluation of corrosion under physiologically relevant conditions.

A notable advantage of pure titanium for biomedical applications lies in its structural tunability, particularly the ability to control porosity. In this regard, additive manufacturing (aka. 3D printing) offers significant potential for the fabrication of titanium scaffolds with complex geometries, high surface area-to-volume ratios, increased surface roughness, and controlled pore structures. Such features not only enable mechanical properties to be tailored to match natural bones but also strongly influence corrosion behavior in physiological environments [27]. Nevertheless, most studies on 3D porous titanium for implants have predominantly focused on alloys rather than commercially pure titanium [14,28]. For example, Dabrowski et al. demonstrated that highly porous titanium scaffolds exhibit mechanical properties and porosities comparable to cancellous bone, supporting their orthopedic application [29]. However, their corrosion performance remains poorly understood. Similarly, Longhitano et al. investigated anodized 3D titanium alloy scaffolds and reported enhanced corrosion resistance, with stability maintained after 168 h of immersion and superior protection relative to non-anodized counterparts [24].

Another important factor determining the clinical success of titanium-based implants is their long-term durability, which governs structural integrity and functional reliability under physiological conditions. During long-term implantation, continuous interactions between the biomaterial surface and the surrounding biological environment can significantly affect corrosion processes. In particular, the formation of additional protective surface layers during immersion may stabilize the passive oxide film and mitigate corrosion degradation [30]. Furthermore, porous architectures can promote protein adsorption, which may provide an additional barrier against corrosive attack. For instance, Pisarek et al. reported that TiO_2_ layers formed on titanium via chemical etching enhanced albumin adsorption compared to untreated Ti surfaces, offering extra protection against corrosion [31]. Despite these observations, the long-term corrosion behavior of additively manufactured titanium biomaterials remains insufficiently explored. This knowledge gap is particularly significant because the majority of corrosion studies focus on short-term electrochemical testing, which may not adequately reflect the conditions experienced by implants during prolonged service in vivo. Moreover, corrosion behavior in complex biological environments is influenced by multiple interacting factors, including proteins, inorganic ions, and the evolution of surface films over time, making long-term investigations essential for a realistic assessment of implant performance.

In this work, we present a comprehensive evaluation of the corrosion behavior of unmodified and TiO_2_-modified three-dimensional titanium scaffolds fabricated by Powder Bed Fusion with Laser Beam (PBF-LB) and subsequently modified by anodic oxidation. The resulting hierarchical micro- and nanostructured surfaces were investigated in corrosion media of increasing physiological complexity, with particular emphasis on prolonged immersion effects. Unlike most previous studies, which have been limited to short-term electrochemical characterization, the present work systematically examines the evolution of corrosion resistance over extended exposure periods in biologically relevant environments. This approach provides a more realistic assessment of the durability and electrochemical stability of titanium biomaterials intended for long-term implantation. To the best of our knowledge, this study provides one of the first comprehensive evaluations of the time-dependent corrosion behavior of anodized additively manufactured titanium scaffolds, extending current knowledge beyond the short-term corrosion assessments reported previously. The findings provide new insights into the evolution and stability of anodized surfaces under prolonged exposure to physiologically relevant conditions and highlight the beneficial role of nanostructured oxide layers in maintaining electrochemical stability over extended periods. By considering the combined effects of scaffold architecture, surface modification, and time-dependent corrosion behavior, this study contributes to the development of more durable and reliable titanium-based implants for bone regeneration applications.

## 2. Results and Discussion

### 2.1. Impact of Corrosion Conditions on Corrosion Resistance

The main factors contributing to corrosion in body fluids include sodium and chloride ions, as well as proteins and amino acids [32]. Due to the complexity of such environments, precise replication is challenging. In this study, the corrosion behavior of unmodified and modified anodized titanium scaffolds was investigated in four media of increasing complexity: 0.9 wt.% NaCl aqueous solution, PBS, SBF, and culture medium supplemented with FBS.

#### 2.1.1. OCP and Tafel Plots

**Figure 1:**
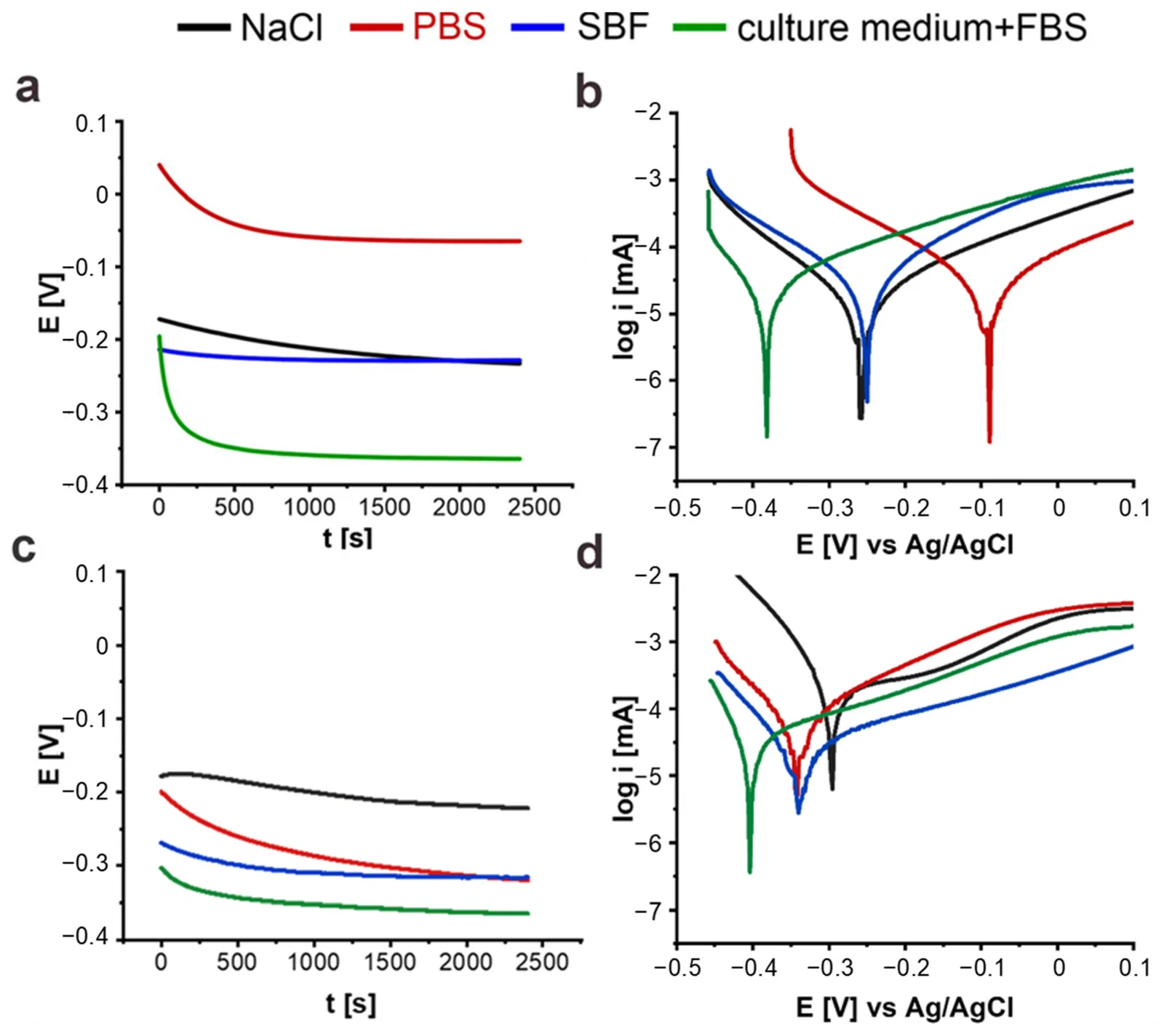
Comparison of the open-circuit potential curves (**a**,**c**) and Tafel plots (**b**,**d**) for representative examples of non-anodized (**a**,**b**) and anodized (**c**,**d**) 3D Ti scaffolds in different corrosive media.

Figure 1a–d presents the open-circuit potential curves and Tafel polarization plots for each electrolyte, comparing non-anodized (Figure 1a,b) and anodized (Figure 1c,d) scaffold samples. Slight fluctuations in potential were observed during the measurements, indicating the dynamic stability of the tested systems. The OCP curves (Figure 1a,c) demonstrate that the protective oxide layer remained intact and passive for the unmodified scaffolds and nanostructured for anodized ones. The negligible decreases in open-circuit potential over time for both non-anodized and anodized Ti scaffolds in NaCl, PBS, SBF, and culture medium with FBS indicate only a minor reduction in the stability of the native oxide layer (non-anodized samples) and the nanostructured oxide layer (anodized samples). This small negative shift reflects a slightly increased tendency for surface reactions or corrosion, as the TiO_2_ film is partially destabilized by ions present in the electrolytes. Preliminary corrosion potentials were determined from OCP measurements by averaging the stabilized potential values, which were in good agreement with the corrosion potentials extracted from the Tafel curves.

For the non-anodized samples, the Tafel curves exhibited similar shapes across all tested corrosive media, suggesting that the corrosion mechanism remained unchanged (Figure 1b). In contrast, for the anodized substrates in NaCl solution, the curve shape differed significantly (Figure 1d). The anodic branch of the curve indicates metal dissolution, as evidenced by the current increase at −0.25 V, followed by a passivation region, where the current remained stable between −0.2 V and −0.1 V. These findings are consistent with the results reported by Loto et al. [33].

From the recorded Tafel curves, the corrosion potential and current density values were determined. Using these data, the corrosion rate and polarization resistance were calculated, as summarized in Table 1. The detailed calculation procedure is provided in the Supplementary Materials (Section S1.2). The reported values are presented as the mean of three independent measurements, with error bars representing the corresponding standard deviation. For the non-anodized scaffolds, the current density and corrosion rate were calculated using the geometric surface area. However, anodization produces a nanoporous TiO_2_ layer, which is expected to increase the accessible surface area compared with the nominal geometric area. To provide a first-order estimate of potential increase in surface area associated with the nanoporous morphology, an approximate geometrical calculation was performed using a simplified cylindrical pore model (the calculation equation is provided in Equation (S3) in the Supplementary Materials). The model parameters were experimentally determined from SEM analysis and ImageJ (version 1.54) image analysis. Specifically, a pore diameter of 75 ± 4 nm and a pore depth of 2.2 ± 0.1 µm were determined from top-view and cross-sectional SEM images, respectively, while a surface porosity of approximately 20% was determined from ImageJ analysis of the SEM image shown in Figure S1 (Supplementary Materials). Based on these experimentally determined parameters, the additional surface area associated with the nanoporous morphology was estimated to be approximately 222.97 mm^2^. Accordingly, the calculated surface area increased from the nominal geometric area of 997.53 mm^2^ to approximately 1220.50 mm^2^, corresponding to an estimated increase of about 22% after anodization. This calculation should be regarded as an approximate geometrical estimation rather than a direct determination of the specific surface area or the electrochemically accessible surface area under corrosion-testing conditions.

For the anodized samples, an increase in the complexity of the corrosive medium resulted in a corrosion potential shift towards more cathodic values. At the same time, a decrease in the corrosion current density was observed, indicating an overall improvement in the corrosion resistance of the anodized samples. In contrast, the j_corr_ values for the non-anodized substrates remained nearly constant across all tested electrolytes. A comparison of the tested materials revealed that the anodized substrates exhibited significantly higher corrosion current densities in NaCl and PBS solutions compared to the non-anodized ones. Consequently, the anodized samples also showed considerably higher corrosion rates in these media, suggesting that the passive layer formed on non-anodized substrates provided more effective protection than that on anodized materials. The different corrosion behavior observed in NaCl and PBS can be attributed to their distinct chemical compositions. Although both media contain chloride ions that may destabilize passive oxide layers, PBS additionally contains phosphate and other buffering ions that can interact with the titanium oxide surface, promoting passive film stability and potentially enhancing corrosion resistance compared with a simple NaCl environment [34,35].

As the ion concentration of the solution increased (e.g., in SBF and cell culture medium supplemented with FBS), the differences in corrosion rate and polarization resistance between anodized and non-anodized substrates diminished, indicating comparable corrosion resistance among the tested materials. Notably, the highest polarization resistance was observed for the non-anodized substrates in NaCl solution, which corresponded to the lowest corrosion rate.

The findings reveal that the composition of the corrosive medium exerts a much greater influence on the corrosion resistance of anodized materials than on non-anodized ones. The nanostructured oxide layer promotes the adsorption of medium components onto the material surface, which enhances corrosion resistance as the electrolyte complexity increases. In contrast, the smooth surface of non-anodized substrates limits adsorption, resulting in relatively consistent corrosion resistance across the different media. Furthermore, as the diversity of ions in the solution increases, the disparity in corrosion resistance between anodized and non-anodized samples diminishes. It should be noted that the corrosion rate values obtained for the anodized substrates should be considered to be approximate, as the surface area correction was based on an estimation derived from SEM micrographs rather than a direct measurement of the electrochemically active surface area. Furthermore, deviations from the idealized pore structure, including variations in pore geometry, pore depth, and the possible presence of cracks or defects within the oxide layer, may influence the actual surface area.

According to the literature, the corrosion rate for metallic implants should remain below 0.25 µm year^−1^ [36], with reported values of approximately 0.17 µm year^−1^ in NaCl and 0.24 µm year^−1^ in the SBF solution [37]. In our study, comparable values were achieved in all electrolytes, except for the anodized samples in NaCl. These results are nevertheless promising and may support potential use of such materials as alternatives to currently employed implants, such as Ti-6Al-4V, while reducing possible adverse effects.

#### 2.1.2. Electrochemical Impedance Spectroscopy

**Figure 2:**
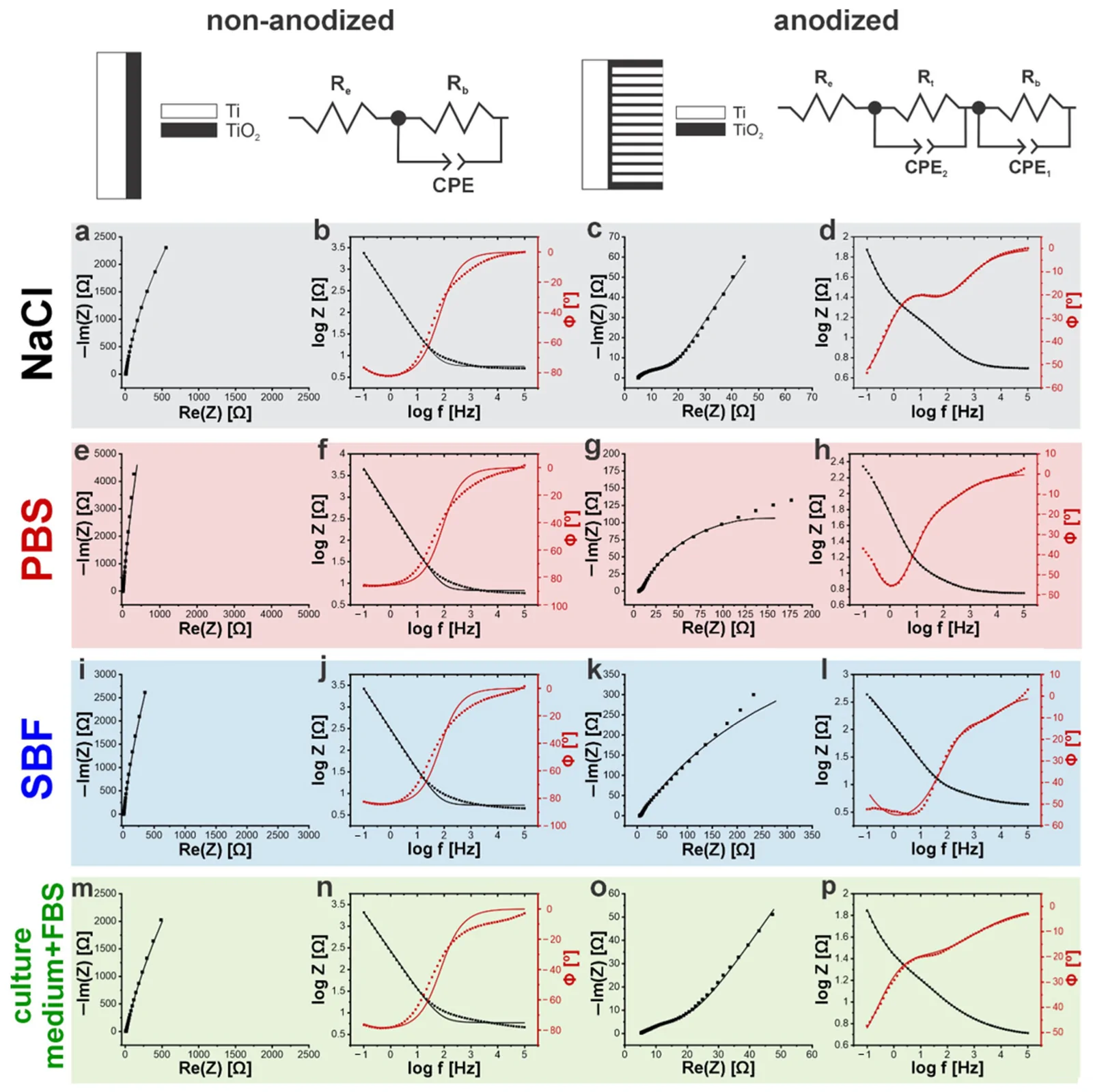
Nyquist (**a**,**c**,**e,g**,**i**,**k**,**m**,**o**) and Bode (**b**,**d**,**f**,**h**,**j**,**l**,**n**,**p**) plots for non-anodized (**a**,**b**,**e**,**f**,**i**,**j**,**m**,**n**) and anodized (**c,d**,**g**,**h**,**k**,**l**,**o**,**p**) samples in NaCl (**a**–**d**), PBS (**e**–**h**), SBF (**i**–**l**), and cell culture media with FBS (**m**–**p**). The equivalent circuits shown at the top were used to fit the respective EIS data. R_e_—electrolyte resistance; R_b_—barrier oxide layer resistance; R_t_—anodic outer oxide layer resistance; CPE—constant phase element of the passive layer; CPE_1_—constant phase element of the barrier oxide layer; CPE_2_—constant phase element of the anodic outer oxide layer.

To further elucidate the corrosion behavior of the tested materials in different electrolytes, EIS measurements were conducted. A comparison of the recorded Nyquist plots for anodized and non-anodized samples in various corrosive media is presented in Figure S2 (Supplementary Materials). For the non-anodized substrates, the Nyquist plots showed a single capacitive semicircle, characteristic of a passive oxide layer. In contrast, the anodized samples exhibited an additional semicircle at higher frequencies, indicative of a more complex and protective surface layer. The presence of this second semicircle reflects improved charge-transfer resistance, suggesting that the anodized layer enhances the material’s ability to resist corrosion processes. The experimental data were analyzed using equivalent circuit models, following the approach proposed by Yu et al. [38]. The quality of the fitting procedure was assessed using the χ^2^ parameter, which evaluates the agreement between the experimental impedance spectra and the proposed equivalent circuit model. In accordance with the approach described by Tribollet et al. [39], equivalent circuit selection should consider not only the quality of the numerical fit but also the physical and chemical processes occurring at the interface. Accordingly, different equivalent circuits were used for the non-anodized and anodized samples to reflect their distinct surface structures and electrochemical responses. For the non-anodized substrates, the spectra were best fitted with a circuit composed of a non-ideal capacitor (constant phase element, CPE) and two resistors (Figure 2). The first resistor (R_b_) corresponds to the resistance of the passive oxide layer, while the second one (R_e_) represents the resistance of the electrolyte. The use of constant phase elements instead of ideal capacitors was selected to account for the non-ideal electrochemical behavior associated with surface heterogeneity and porous oxide architecture [40]. For the anodized samples, a more complex circuit was required, consisting of three resistors and two CPE elements (Figure 2). Specifically, R_b_ represents the resistance of the barrier oxide layer at the pore bottoms, R_t_ corresponds to the resistance of the outer titanium dioxide layer, and R_e_ denotes the electrolyte resistance. The two CPEs were assigned as follows: CPE_1_ to the capacitance of the barrier layer and CPE_2_ to the capacitance of the outer oxide layer. 

Figure 2 summarizes the Nyquist and Bode plots obtained for non-anodized and anodized Ti scaffolds exposed to different corrosive media, along with the corresponding fitted equivalent circuits. For non-anodized substrates (Figure 2a,e,i,m), the Nyquist plots exhibit a nearly linear response with a slope approaching ±1, indicating predominantly capacitive behavior associated with the passive film formation on the titanium surface upon exposure to air. Similar observations have been reported by Longhitano et al. [24]. In contrast, the Bode plots for anodized substrates (Figure 2c,g,k,o) reveal the presence of two distinct time constants, indicating a duplex oxide structure consisting of an inner compact barrier oxide layer and an outer porous oxide layer. The existence of these two electrochemical contributions is further confirmed by the fitted equivalent circuit models, which account for separate interfacial processes occurring at different time scales. Moreover, the anodized samples immersed in the NaCl solution and cell culture medium exhibited pronounced capacitive semicircles in the Nyquist plots, indicative of improved corrosion protection and enhanced stability of the passive layer.

Based on the fitted equivalent circuits, the characteristic electrochemical parameters were determined for both the non-anodized (Table 2) and anodized samples (Table 3).

For the non-anodized samples, the low electrolyte resistance (R_e_) observed in each corrosive medium (Table 2) indicates their purity and the absence of corrosion products. The α parameter, being close to unity, confirms the presence of a smooth, passive oxide layer on the studied scaffolds. The consistent CPE capacitance values across the different media suggest a uniform passive layer thickness and minimal adsorption of medium components onto the surface. The highest resistance (R_b_) was recorded in PBS, although these measurements showed greater variability, resulting in higher uncertainty. However, the highest value of *R_b_*, together with the lowest CPE value, indicates the most resistive and stable oxide layer, reflecting improved corrosion resistance. In contrast, the measurements in NaCl solution exhibited the lowest resistance, underscoring its strong corrosive effect. The results indicate that for non-anodized samples, the influence of the medium’s composition on corrosion resistance remains relatively minor.

Low electrolyte resistance values observed across all tested media confirm their purity and the absence of corrosion products. The increased capacitance of CPE_1_ for NaCl solution and cell culture medium supplemented with FBS is likely due to protein adsorption and salt crystallization on the material surface, as supported by similar R_b_ values. The barrier oxide layer in SBF exhibited significantly higher resistance than in PBS, probably due to the higher ion concentration in SBF, which reduces corrosion by promoting surface adsorption. The α_1_ values around 0.8 indicate the presence of a smooth passive oxide layer at the pore bottoms. The nanoporous oxide layer showed lower resistance than the barrier layer, as the isolated pore bottoms increase the overall resistance. An α_2_ value of approximately 0.6 reflects pronounced surface heterogeneity and porosity of the TiO_2_ layer, leading to non-ideal capacitive behavior. This response arises from a distribution of time constants associated with variations in pore geometry and electrolyte accessibility, resulting in a spatially distributed interfacial impedance across the internal surfaces of the electrode [41]. The lower CPE_2_ capacitance measured in SBF compared to other solutions suggests the formation of a thinner oxide layer, which explains the lower R_t_ value. These findings are consistent with those reported by Zhu et al., who demonstrated that pure Zn exhibits the lowest corrosion rate in cell culture media and the highest in a 0.9% NaCl solution [42]. The lack of buffering capacity and the presence of aggressive chloride ions lead to an increase in pH due to hydroxide ion accumulation. In contrast, bicarbonate and phosphate ions in SBF and cell culture medium facilitate the formation of insoluble corrosion products that effectively inhibit pitting corrosion.

EIS measurements confirmed that a porous oxide layer was successfully formed on the surface of the anodized substrates, with resistance strongly dependent on the ions present in the solution. The barrier layer displayed significantly higher resistance than the nanostructured oxide layer, indicating that the passive layer plays a dominant role in the overall corrosion resistance of the tested materials. For both anodized and non-anodized substrates, the immersion time in electrolyte solutions was not sufficient to initiate notable corrosion. Nevertheless, under the tested conditions, the non-anodized samples exhibited slightly better resistance compared to the anodized ones. Furthermore, the results confirmed that the electrolyte complexity has a pronounced impact on corrosion behavior, particularly for nanostructured samples.

### 2.2. Time-Dependent Corrosion Protection

When implantable materials are considered, it is crucial to assess their long-term behavior under physiological conditions. Therefore, we conducted prolonged corrosion tests in a simulated body environment. The experiments were performed at 37 °C in a culture medium supplemented with 10% FBS over a period of 4 weeks. The electrolyte was selected based on findings from the previous chapter, which demonstrated that electrolyte complexity significantly influences corrosion behavior, particularly in nanostructured materials. As the most chemically complex medium investigated, it provides the closest approximation to physiological conditions, enabling a more representative evaluation of corrosion performance.

#### 2.2.1. OCP and Tafel Plots

**Figure 3:**
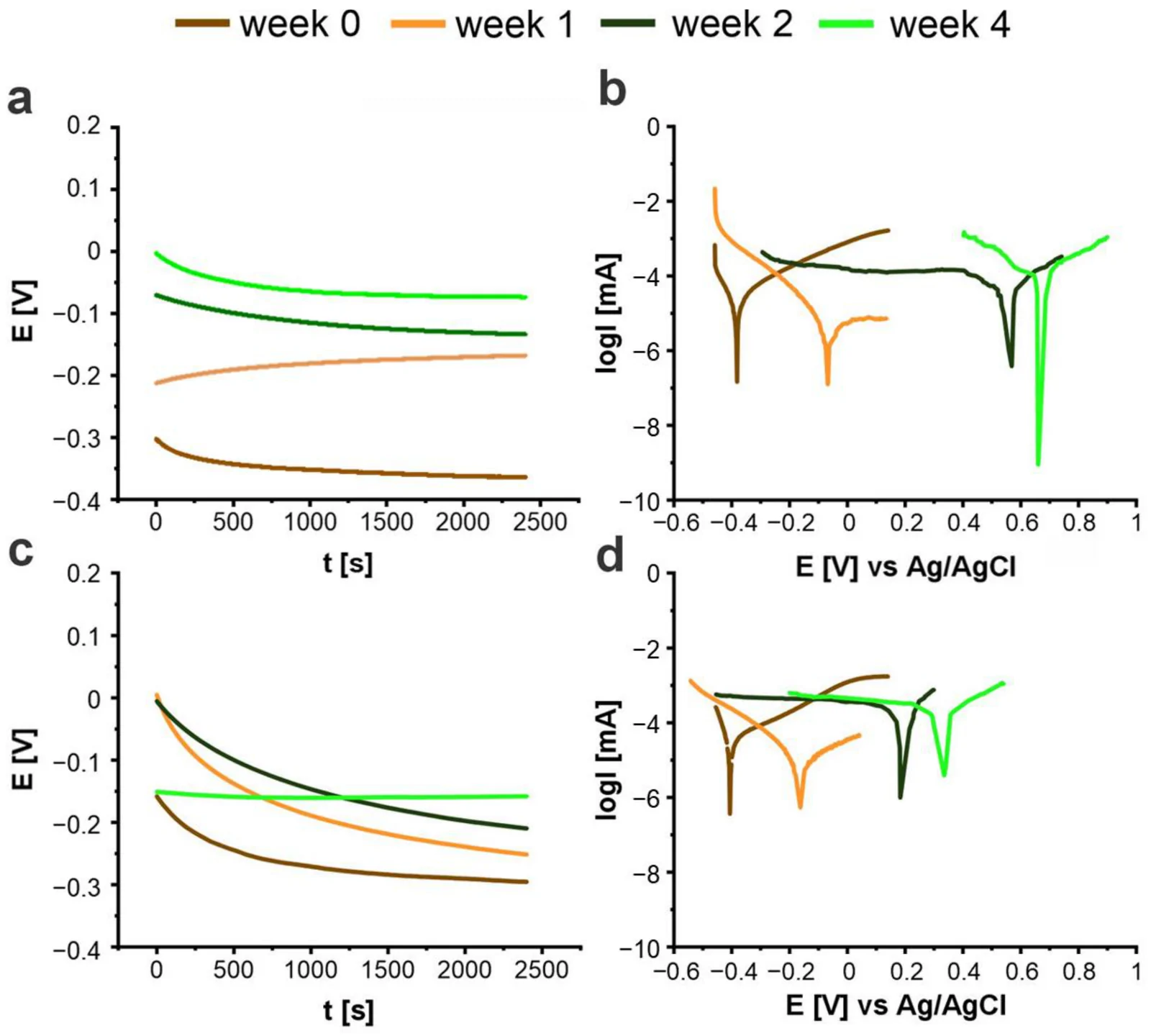
Comparison of the open-circuit potential (OCP) values (**a**,**c**) and recorded Tafel curves (**b**,**d**) for representative examples of non-anodized (**a**,**b**) and anodized (**c**,**d**) samples after different immersion times in culture medium supplemented with 10% FBS.

Figure 3 presents the measured open-circuit potential values (Figure 3a,c) and the Tafel curves (Figure 3b,d) after different immersion times in the corrosive medium for both non-anodized and anodized samples.

Only minor fluctuations in the open-circuit potential were observed, indicating a relatively stable electrochemical response of the system throughout the measurement period. After two weeks of incubation, noticeable changes appeared in the Tafel curves for both non-anodized and anodized samples. Specifically, a reduction in the slope of the cathodic branch was detected, suggesting a modification in the corrosion mechanism [43]. This behavior may be attributed to the formation of an additional protective layer on the material surface.

For anodized samples, prolonged immersion in the corrosive medium resulted in an anodic shift in potential and an increase in corrosion current density, pointing to a gradual thinning or destabilization of the oxide layer (Table 4). During the first week of immersion, j_corr_ values for both anodized and non-anodized samples remained low, which can be attributed to the presence of a protective TiO_2_ film that initially limited ionic transport. In non-anodized substrates, a pronounced decrease in corrosion current density was observed after the first week, suggesting that adsorption of serum proteins and incorporation of phosphate or bicarbonate ions promoted the stabilization and thickening of the passive oxide [44,45]. After two weeks of incubation, a marked increase in j_corr_ was observed for both anodized and non-anodized samples. This indicates a deterioration in corrosion resistance, most likely caused by partial breakdown of the oxide layer. This effect was more pronounced in non-anodized samples, where the thinner native oxide offered weaker protection. Such degradation could arise from competitive adsorption of chloride ions, which locally disrupt the passive layer, together with protein degradation products that interfere with oxide stability [46]. In anodized samples, the higher surface area and porosity of the nanostructured oxide likely accelerated ion exchange processes, leading to enhanced susceptibility to localized corrosion. Additionally, with prolonged immersion in the corrosive medium, the potential obtained from the OCP measurements deviates significantly from the corrosion potential derived from the Tafel plots. The Tafel-derived E_corr_ reflects the surface behavior under polarization, during which the passive layer may undergo modification, whereas OCP represents the potential of the surface prior to any perturbation. This behavior may be attributed to enhanced protein adsorption during polarization in the electrolyte solution. Adsorbed species at the metal–liquid interface strongly influence electrochemical corrosion. Proteins can reduce metal ion release by forming a stable adsorbed layer that blocks electrochemical dissolution or, alternatively, form metal–protein complexes (chelates) that promote oxide dissolution [30]. Divalent metal ions present in body fluids can adsorb onto the surface via electrostatic interactions, increasing the positive charge of the oxide layer and thereby enhancing the attraction of negatively charged proteins. These proteins preferentially adsorb at cathodic sites and compete with aggressive ions such as Cl^−^ [47]. Non-anodized samples exhibit greater divergence between OCP and E_corr_ due to the limited thickness and protective capacity of their native oxide layer, in contrast to the thicker, porous oxide layers formed by anodization. Han et al. reported that L-arginine and L-lysine inhibit corrosion through chemisorption (coordination via N/O atoms) and physisorption (electrostatic attraction between protonated molecules and Cl^−^ ions) [48]. Protonation leads to the formation of positively charged species (R^+^), which increases the OH^−^ ion concentration and suppresses the cathodic reaction. This behavior is closely related to the isoelectric point (pI): amino acids with a pI higher than the solution pH become positively charged and readily adsorb onto the surface, whereas those with a lower pI, such as L-histidine (pI = 7.59), tend to be negatively charged, promoting competitive adsorption with chloride ions. All of these amino acids were present in the electrolyte, which may explain the differences observed in the corrosion parameters. An additional contributing factor may be the thinning of the oxide layer during polarization. This process predominantly occurs under cathodic polarization, where the applied negative potential creates reducing conditions that destabilize the oxide film, leading to its breakdown and partial dissolution [49]. Therefore, the discrepancy observed during prolonged exposure can be primarily attributed to time-dependent alterations of the passive oxide layer, competitive adsorption of proteins and aggressive ions, and the possible initiation of localized corrosion [50]. Based on the presented results, it can be hypothesized that physicochemical changes occurred on the oxide layer surface after two weeks of immersion, subsequently influencing the corrosion mechanism; however, its detailed characteristics still require thorough evaluation.

#### 2.2.2. Electrochemical Impedance Spectroscopy

To gain deeper insight into the mechanisms governing the corrosion of the tested materials, electrochemical impedance spectroscopy measurements were conducted after respective incubation times. Nyquist plots for anodized and non-anodized samples after different immersion times in a corrosive medium are shown in Figure S3 (Supplementary Materials). For non-anodized substrates, the Nyquist plots displayed a single semicircle, corresponding to the passive oxide layer on the surface. The gradual increase in impedance during the first week suggests progressive stabilization of the oxide layer, likely facilitated by adsorption of medium components such as phosphate and bicarbonate ions, which can reinforce the passive film. Minor fluctuations in subsequent weeks indicate that the passive layer remained largely intact, providing sustained corrosion resistance. In contrast, anodized samples exhibited an additional capacitive semicircle at high frequencies, indicative of the nanostructured outer oxide layer. The presence of this layer increases the surface area and introduces isolated pore bottoms, which initially allow localized ion exchange but also provide additional sites for biomolecule adsorption. After two weeks of immersion, a pronounced increase in impedance was observed, reflecting the formation of a denser, more stable protective layer. This behavior can be attributed to the combined effects of oxide restructuring and adsorption of biomolecules such as L-histidine, which promote the formation of a continuous, corrosion-inhibiting film on the metal surface [37]. Overall, the EIS results indicate that both the passive and nanostructured oxide layers interact dynamically with the ions and biomolecules in the medium, modulating the corrosion behavior and enhancing the stability of the materials during prolonged exposure.

**Figure 4:**
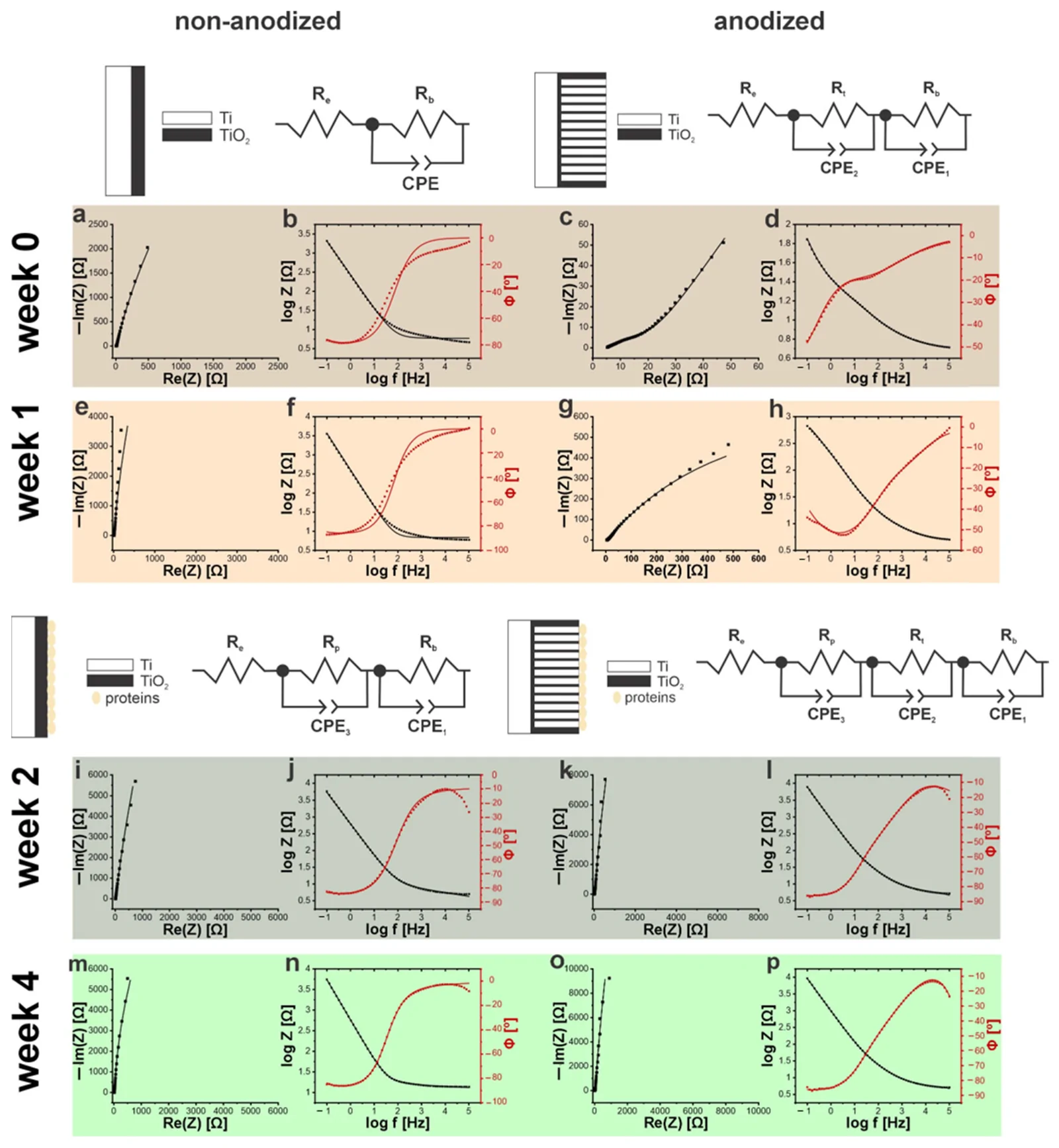
Nyquist and Bode plots recorded for non-anodized (**a**,**b**,**e**,**f**,**i**,**j**,**m**,**n**) and anodized (**c**,**d**,**g**,**h**,**k**,**l**,**o**,**p**) samples after week 0 (**a**–**d**), week 1 (**e**–**h**), week 2 (**i**–**l**), and week 4 (**m**–**p**). The equivalent circuits shown at the top were used to fit the respective EIS data. R_e_—electrolyte resistance; R_b_—barrier oxide layer resistance; R_t_—anodic outer oxide layer resistance; R_p_—protein layer resistance; CPE_1_—constant phase element for the barrier oxide layer; CPE_2_–constant phase element for the anodic outer oxide layer; CPE_3_–constant phase element for the adsorbed protein layer.

The experimental results were initially modeled using an equivalent circuit consisting of a constant phase element and two resistors (R_e_ and R_b_). As immersion progressed in the protein-containing electrolyte, the evolution of the electrode/electrolyte interface resulted in a more complex electrochemical response, requiring a modified equivalent circuit to accurately describe the interfacial processes. During the second and fourth weeks, the observed increase in impedance indicated the adsorption of proteins onto the surface of both anodized and non-anodized samples [38]. To account for this additional interfacial process, the equivalent circuit was refined by adding a second CPE and a parallel resistor R_p_, representing the formation of an additional surface layer that impedes charge transfer. The quality of the fitting was assessed using the χ^2^ parameter, which confirmed a good agreement between the experimental impedance spectra and the proposed equivalent circuit models. The improved fit confirms that the interaction of biomolecules with the oxide surface leads to the development of a protective, adsorption-mediated layer, which contributes to enhanced corrosion resistance (Figure 4).

Figure 4 shows the recorded Nyquist and Bode plots for non-anodized and anodized samples, together with the corresponding fitted equivalent circuits.

For anodized samples, the impedance increased with prolonged incubation in the corrosive medium, with the most significant change observed between the first and second weeks. This behavior is attributed to the formation of an additional protective layer, most likely consisting of adsorbed proteins. In contrast, for non-anodized substrates, the impedance increase after the second week was considerably smaller, indicating that although a protein layer also formed, it was thinner and less effective at providing corrosion resistance compared to that on the nanostructured oxide surface. The presence of this layer is further supported by the phase angle shift observed in the Bode plots for the second week of incubation for both types of materials. Table 5 shows the values of equivalent circuit elements after different immersion times in the corrosive medium for non-anodized and anodized materials. After two weeks of immersion, non-modified substrates exhibited a marked decrease in electrolyte resistance and an increase in passive-layer resistance. After an additional two weeks, R_e_ increased while R_b_ decreased due to damage to the protective layer. This interpretation is further supported by the decrease in CPE_1_, corresponding to the barrier layer. The low α values associated with the protein layer indicate uneven surface coverage. Between the second and fourth weeks of incubation, reductions in both R_p_ and CPE_3_ suggested changes in the protective layer. The interpretation of capacitance values obtained from EIS should be considered carefully, as the measured interfacial response may include contributions from both the oxide layer and the electrochemical double layer [51]. Therefore, changes in capacitance values are interpreted here as variations in the electrochemical characteristics of the surface film/interface rather than as direct evidence of changes in oxide-layer thickness. For anodized samples, extended incubation in the corrosive medium resulted in a general decrease in electrolyte resistance, reflecting reduced solution penetration through the oxide layers. After two weeks, a significant increase in the barrier layer resistance was observed, most likely due to the formation of an additional protective layer of adsorbed proteins on the surface. This was accompanied by a marked reduction in electrolyte resistance. After four weeks, further increases in both barrier-layer and protein-layer resistance indicated greater accumulation of proteins on the surface. The fitted capacitance values indicate that the adsorbed protein layer and the passive oxide layer exhibit distinct electrochemical responses. The stability of the barrier layer was corroborated by consistently high α values throughout the immersion period. Over time, the decrease in the capacitance of the passive layer indicates changes in the electrochemical characteristics of the passive film during prolonged exposure. Meanwhile, the minimal variations in both the capacitance and the resistance values of the nanostructured oxide layer suggest that this layer maintained stable electrochemical behavior and remained protective under the corrosive environment.

Overall, the results indicate that the corrosion resistance of anodized titanium substrates improves with prolonged immersion in the electrolyte. Compared to non-modified substrates, the anodized materials exhibit markedly superior time-dependent electrochemical stability in the corrosive environment, with the beneficial effects becoming more pronounced over extended exposure times. This enhanced time-dependent stability can be attributed to the strong affinity of the nanostructured titanium dioxide surface for components of the medium, which promotes the gradual formation of a more stable and protective interfacial layer during prolonged immersion. This behavior highlights the importance of evaluating long-term corrosion performance in complex, bio-relevant environments, as time-dependent interactions between the material surface and the surrounding medium can significantly influence degradation processes and electrochemical stability.

#### 2.2.3. Scanning Electron Microscopy Analysis

**Figure 5:**
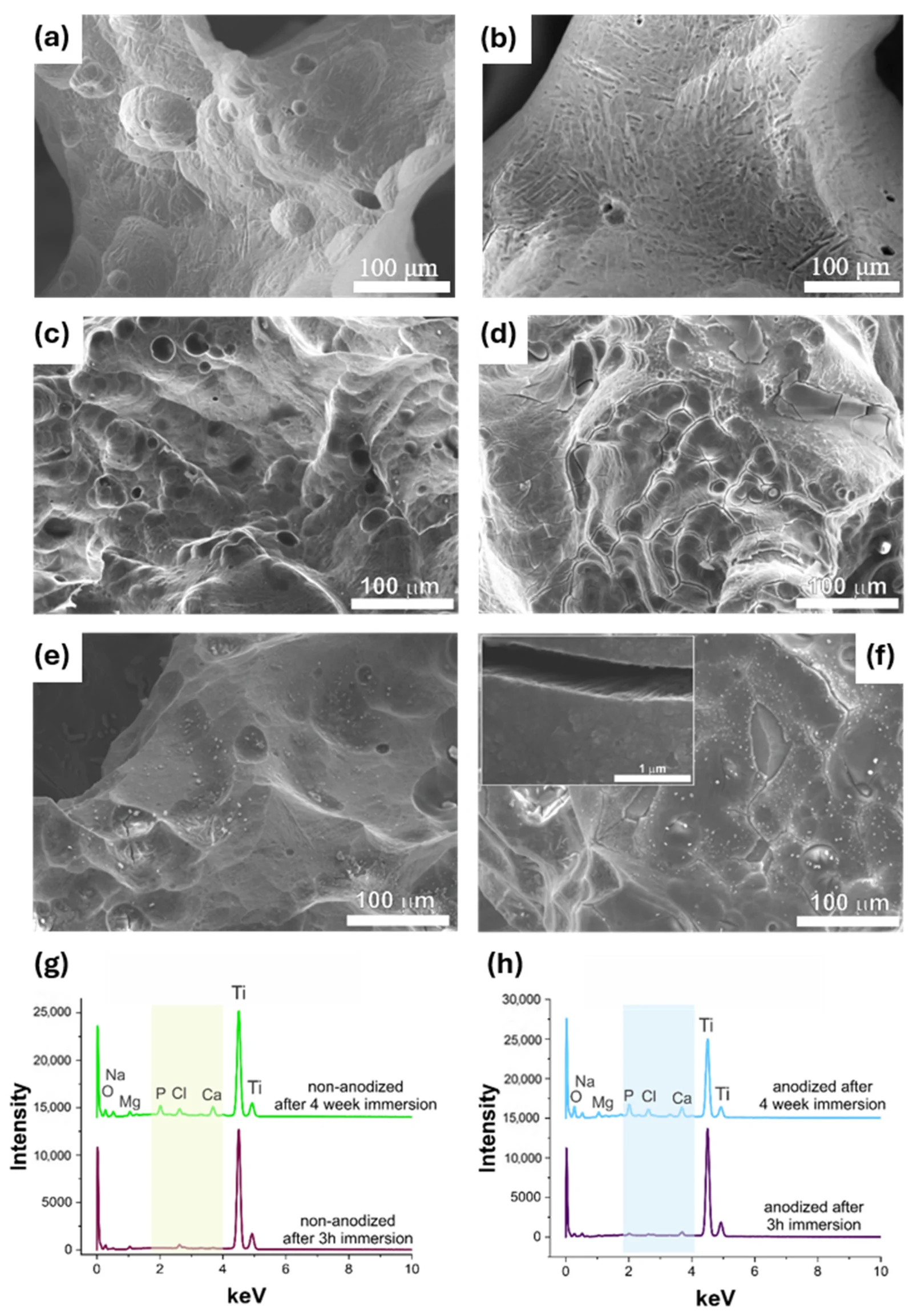
SEM micrographs of non-anodized (**a**) and anodized (**b**) samples before immersion, non-anodized (**c**) and anodized (**d**) samples at week 0 in the culture medium, and non-anodized (**e**) and anodized (**f**) samples after 4 weeks of immersion in the culture medium. The inset in (**f**) shows a cross-section of the oxide layer for the anodized sample after 4 weeks. EDS spectra of non-anodized and anodized samples after short (**g**) and prolonged (**h**) immersion in the culture medium.

To assess surface morphology changes, SEM images were acquired for both non-anodized and anodized samples before and after short and prolonged incubation in the culture medium (Figure 5).

SEM images of anodized samples after 3 h of immersion in the electrolyte (week 0) reveal a porous layer with surface cracks, indicating the formation of a nanostructured oxide layer. In contrast, non-anodized substrates exhibited a smooth surface, consistent with the presence of only a naturally occurring passive film. Although the presence of microcracks may provide preferential pathways for electrolyte penetration and localize corrosion initiation, their impact on corrosion resistance depends on the overall integrity, thickness, and stability of the oxide layer. In the case of anodized titanium scaffolds, the presence of a stable and adherent oxide layer may limit the propagation of corrosion processes by maintaining protective surface properties. Nevertheless, the observed microcracks could represent potential weak points that may affect performance under prolonged exposure to physiological environments. After four weeks in the corrosive medium, brighter surface features appeared, attributed to adsorbed components from the medium. These features were more pronounced on the anodized samples, whose nanostructured oxide layer also displayed increased surface roughness due to enhanced adsorption.

The corresponding EDS analyses (Figure 5g,h) provide the elemental composition of the materials, while quantitative results are summarized in Table 6.

After three hours of immersion in the solution, both anodized and non-anodized substrates exhibited the presence of elements in addition to titanium and oxygen. The reported values represent the average composition obtained from multiple analyzed areas on the sample surface; however, the relatively high variability reflects the heterogeneous nature of the biological interface formed during immersion, including the non-uniform adsorption of proteins and biomolecules The notable high carbon content indicated protein adsorption on the surface, while magnesium, phosphorus, and potassium, originating from the medium, were detected exclusively on the anodized samples. The comparable carbon levels on both substrates suggest similar protein adsorption at this early stage, consistent with their similar corrosion resistance. After four weeks, the elevated carbon content confirmed the development of a protective protein layer. Both substrate types also exhibited higher levels of adsorbed elements from the medium, including sodium, phosphorus, potassium, and calcium, as well as traces of sulfur and chlorine. These findings align with the improved corrosion resistance observed in EIS measurements. The observed behavior can be explained by the complex interactions between the anodic TiO_2_ surface, dissolved ions, and biomolecules present in the physiological electrolyte. Divalent ions can interact with the oxide surface and modify its local charge, thereby promoting the adsorption of proteins through electrostatic and other surface interactions [52]. The resulting protein layer can act as an additional barrier to the transport of aggressive species, while adsorbed biomolecules compete with Cl^−^ ions for available surface sites. Consequently, the combined effects of ion-mediated protein adsorption and the protective nanostructured TiO_2_ layer contribute to limiting electrochemical dissolution and maintaining the corrosion resistance of the anodized scaffolds during prolonged exposure. Importantly, the greater extent of protein adsorption on anodized surfaces highlights the role of the nanostructured oxide layer in enhancing the long-term corrosion resistance of anodized materials under prolonged exposure to a corrosive environment.

However, this study is subject to several practical constraints that are worth noting. The reproducibility of the samples shows minor variability, which is reflected in moderate dispersion of the electrochemical measurements. Moreover, the corrosion rate values obtained for the anodized substrates should be considered approximate, as the surface area correction was based on an estimation derived from SEM micrographs rather than a direct determination of the electrochemically active surface area. Furthermore, deviations from the idealized pore structure, including variations in pore geometry, pore depth, and the possible presence of cracks or defects within the oxide layer, may affect the actual accessible surface area and introduce additional uncertainty into the calculated corrosion rates. Taken together, these factors represent inherent experimental challenges rather than fundamental limitations of the approach. Importantly, they do not affect the overall reliability of the observed trends. The results indicate that the synthesized materials demonstrate strong potential for long-term corrosion protection in biomedical environments and offer a robust platform for further surface modification. Although the 4-week test provides valuable information on the initial corrosion behavior and stability of the modified titanium scaffolds, extended studies under prolonged physiological conditions are required to better evaluate long-term degradation mechanisms, surface stability, and potential changes in corrosion resistance over time. Future work will focus on long-duration immersion tests to provide a more comprehensive assessment of the long-term performance and reliability of the developed implant materials. Nevertheless, the present findings are highly relevant from a biomedical perspective, as improving the stability of the titanium surface through anodization may contribute to reducing metal ion release and preserving the structural integrity of implants during long-term service, which are critical factors for the safety and reliability of orthopedic devices. Moreover, maintaining a stable oxide surface under physiologically relevant conditions is essential not only for corrosion protection but also for preserving a favorable implant–tissue interface. Therefore, the demonstrated time-dependent electrochemical stability of anodized additively manufactured titanium scaffolds represents an important step toward the development of more durable bone implant materials.

## 3. Materials and Methods

### 3.1. Preparation of Modified 3D Scaffolds

**Figure 6:**
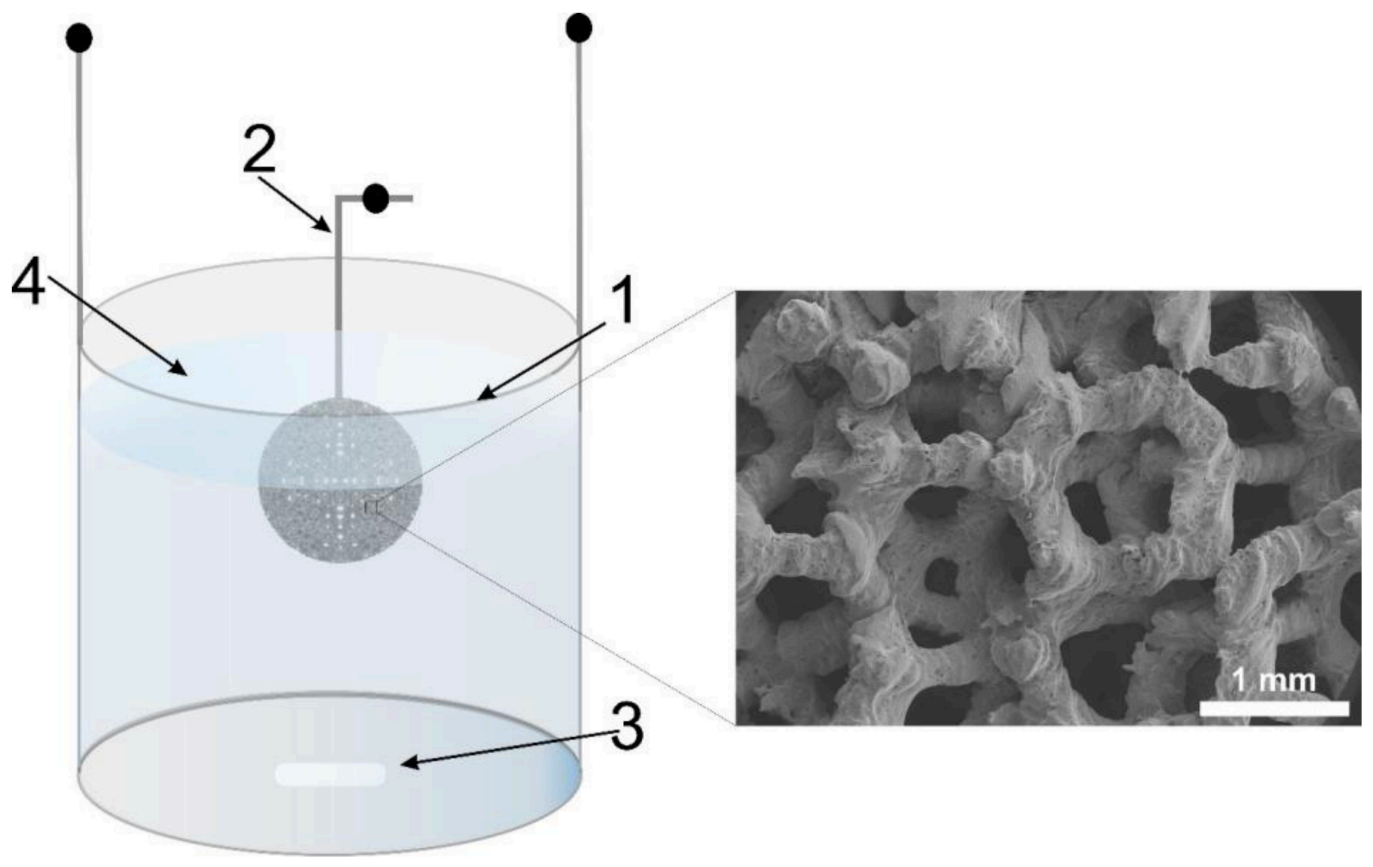
Schematic representation of the anodization setup for 3D titanium scaffolds (1—cylindrical titanium cathode; 2—additive manufactured Ti scaffold; 3—magnetic stirrer; 4—ethylene glycol-based electrolyte).

Three-dimensional titanium scaffolds in the form of a disk (12 mm in diameter, 3 mm in thickness) with pore sizes ranging from 200 to 700 μm and a total surface area of 997.53 mm^2^ were designed based on Laguerre–Voronoi tessellation [53] and manufactured from commercially pure titanium (CP Ti). The graded architecture was designed using Materialise Magics 26.01 software, employing the Magics Structures module in combination with Boolean operations. The scaffolds were fabricated via Powder Bed Fusion with Laser Beam (PBF-LB) using a Realizer SLM50 system (Realizer GmbH/DMG Mori, Borchen, Germany). The manufacturing process was carried out at a laser power of 42 W and a scanning speed of 375 mm s^−1^, with a layer thickness of 25 μm under an inert argon atmosphere. The detailed procedure was described by Idaszek et al. [54]. The scaffold surface area was calculated from CAD models using Materialise Magics software (Materialise NV, version 28.0, Leuven, Belgium). The scaffolds exhibited a random pore distribution, with an open porosity of 75%, determined based on CAD models. Before the anodization, all samples were chemically cleaned by ultrasonic etching for 5 min at room temperature in a mixture of 65% nitric acid, 40% hydrofluoric acid, and distilled water [55]. Subsequently, the samples were thoroughly rinsed with distilled water and dried in air. The non-anodized samples were likewise subjected to the same etching procedure prior to corrosion testing to ensure complete removal of any thermally formed oxide layer generated during the additive manufacturing process. Anodization was carried out at 20 °C using a two-electrode system with stirring at 300 rpm. The scaffold specimen acted as the working anode, whereas a cylindrical, closed-bottom titanium electrode served as the cathode (Figure 6).

Additive manufactured Ti scaffolds were anodized using a previously developed one-step procedure [56,57,58,59]. The optimized anodizing conditions were voltage, 40 V; time, 15 min; and electrolyte composition of 0.38 wt.% ammonium fluoride and 1.97 wt.% water, with ethylene glycol as the solvent. The distance between the electrodes was 14 mm. The electrolyte volume inside the cylindrical cathode was 25 cm^3^. After anodization, the samples were rinsed with deionized water and air-dried. After electrochemical oxidation, nanostructured oxide layers were formed on the 3D Ti scaffolds, with a pore diameter of 75 ± 4 nm and a layer thickness of 2.2 ± 0.1 µm, as determined using field emission scanning electron microscopy (FE-SEM, Hitachi S-4700; Hitachi, Tokyo, Japan) (Figure S1, Supporting Information).

### 3.2. Corrosion Tests

Corrosion behavior of the fabricated structures was evaluated using a Bio-Logic SP-300 potentiostat operated with EC-Lab software version 11.50 (Bio-Logic, Grenoble, France). All measurements were performed at 37 °C ± 1 °C in a standard three-electrode configuration, with non-anodized or anodized titanium scaffolds as the working electrode, a silver/silver chloride electrode (Ag/AgCl, saturated KCl) as the reference electrode, and a platinum mesh as the counter electrode. The open-circuit potential (OCP) was monitored for 40 min to ensure stabilization, after which linear polarization (Tafel analysis) was recorded over a potential range of −450 to 150 mV vs. Ag/AgCl, using a scan rate of 0.1 mV s^−^. From the recorded Tafel curves, the corrosion potential and corrosion current density were determined [60]. Electrochemical impedance spectroscopy (EIS) was conducted at the corrosion potential determined from the Tafel curves over a frequency range of 100 kHz to 100 mHz, with a 10 mV AC amplitude. Measurements were carried out in four progressively complex corrosive environments: 0.9 wt.% sodium chloride solution, pH = 7; phosphate-buffered saline (PBS), pH = 7.3 (Dulbecco’s Phosphate Buffered Saline with MgCl_2_ and CaCl_2_, Sigma-Aldrich, Saint Louis, MI, USA); simulated body fluid (SBF), pH = 7.4; and cell culture medium (Dulbecco’s Modified Eagle’s Medium—high glucose, with 4500 mg L^−1^ glucose, L-glutamine, sodium pyruvate, and sodium bicarbonate, Sigma-Aldrich) supplemented with fetal bovine serum (FBS, Sigma-Aldrich) at a 9:1 ratio, pH = 7.2. The SBF solution was prepared according to the method described by Kokubo et al. [44]. Experimental data were derived from three independent replicates (*n* = 3).

### 3.3. Long-Term Corrosion Tests

The prolonged corrosion resistance of the prepared samples was investigated over a 4-week period. Samples were incubated in cell culture medium (Dulbecco’s Modified Eagle’s Medium—high glucose, with 4500 mg L^−1^ glucose, L-glutamine, sodium pyruvate, and sodium bicarbonate, Sigma-Aldrich) supplemented with 10% fetal bovine serum and maintained in a water bath at 37 °C. Electrochemical measurements were performed after 1, 2, and 4 weeks, with the samples returned to the same medium for continued incubation between tests. Experiments were conducted for three independent replicates (*n* = 3). In this part of the research, the linear polarization measurements (Tafel analysis were conducted within an extended potential range selected between −450 and 900 mV vs. Ag/AgCl, at a scan rate of 0.1 mV s^−1^, to capture both the anodic and cathodic branches of the polarization curves and enable a more comprehensive evaluation of the corrosion behavior.

### 3.4. Characterization of Tested Materials

The surface morphology of the samples was examined using a field emission scanning electron microscope (FE-SEM, Hitachi S-4700, Tokyo, Japan) operating at an acceleration voltage of 20 kV. Elemental surface composition was determined by energy-dispersive X-ray spectroscopy (EDS, Hitachi S-4700, Tokyo, Japan) with a Si(Li) detector with 134 eV resolution and 30 mm^2^ active area. Point analyses were carried out at randomly selected regions on the sample surface.

## 4. Conclusions

This study presents a comprehensive evaluation of the corrosion resistance of 3D titanium scaffolds, both in the non-anodized and the anodized state. A key outcome of this work is the demonstration that the corrosion response of anodized scaffolds (with a nanostructured titanium dioxide layer on top) is strongly dependent on electrolyte composition, highlighting the pronounced role of complex environment–surface interactions in governing electrochemical behavior under physiologically relevant conditions. In contrast, non-anodized samples exhibited lower environmental sensitivity to electrolyte composition and, in the short term, demonstrated comparatively higher corrosion resistance; however, their prolonged performance was inferior. These findings clearly indicate that short-term electrochemical measurements may not adequately reflect the true durability of titanium-based biomaterials under prolonged exposure. Surface modification with titanium dioxide significantly improved corrosion resistance during prolonged exposure, primarily through enhanced protein adsorption. The nanostructured TiO_2_ coating promoted more effective stabilization of the protective film, while the naturally formed oxide layer on non-anodized substrates was thinner and less durable over time. These results highlight the importance of time-dependent surface–medium interactions, including processes such as protein adsorption, in governing long-term electrochemical stability in bio-relevant environments. Overall, this work emphasizes the necessity of systematic long-term corrosion testing in complex, bio-relevant environments, as only such an approach can reliably capture degradation processes relevant to in vivo conditions. The findings also demonstrate the potential of optimizing nanostructured oxide coatings to enhance the long-term electrochemical performance of titanium-based implants. Furthermore, additive manufacturing offers significant advantages, including precise control over scaffold architecture, porosity, and mechanical properties such as Young’s modulus, enabling better matching to the properties of bone tissue. When combined with electrochemical surface modification, this approach provides a promising route for the development of advanced biomaterials that exhibit both corrosion resistance and mechanical compatibility, representing a viable alternative to conventional implant materials.

## Figures and Tables

**Table 1:** Average corrosion parameters obtained from Tafel curve analysis in different corrosive media. E_corr_—corrosion potential, j_corr_—current density at E_corr_, v_corr_—corrosion rate, R_p_—polarization resistance. The reported values represent the average of measurements obtained from three samples.

Electrolyte		OCP [V]	E_corr_ [V]	j_corr_ [μA cm^−2^]	v_corr_ [μm year^−1^]	R_p_ [MΩ cm^2^]
NaCl	Non-anodized	−0.23 ± 0.01	−0.25 ± 0.01	0.0012 ± 0.0001	0.021 ± 0.002	1300 ± 40
	Anodized	−0.22 ± 0.03	−0.29 ± 0.01	0.016 ± 0.007	0.27 ± 0.11	49 ± 12
PBS	Non-anodized	−0.018 ± 0.048	−0.03 ± 0.06	0.0027 ± 0.0007	0.046 ± 0.011	410 ± 80
	Anodized	−0.33 ± 0.01	−0.34 ± 0.05	0.0098 ± 0.0049	0.17 ± 0.08	171 ± 12
SBF	Non-anodized	−0.22 ± 0.03	−0.26 ± 0.05	0.0036 ± 0.0018	0.063 ± 0.031	320 ± 30
	Anodized	−0.32 ± 0.02	−0.35 ± 0.03	0.0031 ± 0.0019	0.053 ± 0.034	549 ± 256
Culture medium + FBS	Non-anodized	−0.32 ± 0.04	−0.39 ± 0.09	0.0047 ± 0.0036	0.081 ± 0.063	700 ± 160
	Anodized	−0.37 ± 0.09	−0.38 ± 0.03	0.0033 ± 0.0016	0.057 ± 0.027	695 ± 134

**Table 2:** Average values of equivalent circuit elements for non-anodized samples in different corrosive media.

Electrolyte	R_e_ [Ω]	CPE [mF s^a−1^]	α	R_b_ [kΩ]	χ^2^
NaCl	5.57 ± 0.05	0.69 ± 0.04	0.94 ± 0.03	14 ± 5	0.93 ± 0.17
PBS	6.66 ± 2.69	0.41 ± 0.09	0.96 ± 0.02	150 ± 50	1.28 ± 0.15
SBF	5.32 ± 0.05	0.55 ± 0.03	0.94 ± 0.01	56 ± 3	0.079 ± 0.76
Culture medium + FBS	5.62 ± 0.29	0.88 ± 0.16	0.86 ± 0.04	25 ± 4	1.78 ± 0.18

**Table 3:** Average values of equivalent circuit elements for anodized samples in different corrosive media.

Electrolyte	R_e_ [Ω]	CPE_1_ [mF s^a−1^]	α_1_	R_b_ [kΩ]	CPE_2_ [mF s^a−1^]	α_2_	R_t_ [Ω]	χ^2^
NaCl	4.6 ± 0.3	25 ± 10	0.73 ± 0.02	1.3 ± 0.3	4.6 ± 0.1	0.58 ± 0.01	14 ± 7	0.011 ± 0.007
PBS	5.6 ± 0.1	3.8 ± 0.2	0.84 ± 0.03	0.6 ± 0.3	4.0 ± 0.5	0.64 ± 0.01	4.1 ± 0.1	0.0075 ± 0.0031
SBF	4.3 ± 0.3	2.7 ± 1.7	0.86 ± 0.02	2.1 ± 0.9	0.06 ± 0.64	0.66 ± 0.03	1.8 ± 0.9	0.043 ± 0.023
Culture medium + FBS	5.6 ± 0.3	28 ± 3	0.77 ± 0.11	1.7 ± 0.5	6.9 ± 4.0	0.48 ± 0.10	22.5 ± 7.5	0.039 ± 0.049

**Table 4:** Average corrosion parameters obtained from the Tafel curve analysis for anodized and non-anodized samples after different immersion times in a corrosive medium. The reported values represent the average of measurements obtained from three samples.

Time of Immersion		OCP [V]	E_corr_ [V]	j_corr_ [μA cm^−2^]
0 week	Non-anodized	−0.32 ± 0.04	−0.39 ± 0.09	0.0047 ± 0.0036
	Anodized	−0.37 ± 0.09	−0.38 ± 0.03	0.0033 ± 0.0016
1 week	Non-anodized	−0.033 ± 0.019	−0.063 ± 0.004	0.00040 ± 0.00011
	Anodized	−0.24 ± 0.10	−0.32 ± 0.15	0.0043 ± 0.0064
2 weeks	Non-anodized	−0.071 ± 0.078	0.59 ± 0.03	0.011 ± 0.002
	Anodized	−0.017 ± 0.034	0.19 ± 0.06	0.012 ± 0.002
4 weeks	Non-anodized	−0.12 ± 0.01	0.65 ± 0.02	0.0055 ± 0.0028
	Anodized	−0.25 ± 0.10	0.31 ± 0.03	0.015 ± 0.0016

**Table 5:** Equivalent circuit parameters obtained after different immersion times in the corrosive medium for non-anodized and anodized materials. The reported values represent the average of measurements obtained from three samples.

	Time of Immersion	R_e_ [Ω]	CPE_1_ [mF s^a−1^]	α_1_	R_b_ [kΩ]	CPE_2_ [mF s^a−1^]	α_2_	R_t_ [Ω]	CPE_3_ [μF s^a−1^]	α_3_	R_p_ [Ω]	χ^2^
Non-anodized	0 week	5.6 ± 0.3	0.88 ± 0.16	0.86 ± 0.04	25 ± 4							0.93 ± 0.17
	1 week	6.8 ± 0.1	0.36 ± 0.07	0.97 ± 0.01	130 ± 10							1.28 ± 0.21
	2 weeks	2.6 ± 1.2	0.25 ± 0.03	0.96 ± 0.01	238 ± 31				0.020 ± 0.007	0.25 ± 0.07	22 ± 1	0.25 ± 0.07
	4 weeks	8.6 ± 3.7	0.24 ± 0.05	0.98 ± 0.01	94 ± 20				0.013 ± 0.009	0.37 ± 0.09	11 ± 3	0.095 ± 0.019
Anodized	0 week	5.6 ± 0.3	28 ± 3	0.77 ± 0.11	1.6 ± 0.5	6.9 ± 4.0	0.48 ± 0.09	23 ± 8				0.039 ± 0.049
	1 week	5.1 ± 0.3	2.5 ± 1.0	0.92 ± 0.04	1.7 ± 0.1	2.7 ± 1.3	0.53 ± 0.08	29 ± 12				0.043 ± 0.043
	2 weeks	0.94 ± 0.40	0.23 ± 0.02	0.96 ± 0.01	344 ± 20	4.6 ± 0.5	0.57 ± 0.01	38 ± 1	0.21 ± 0.09	0.99 ± 0.01	4.4 ± 0.5	0.022 ± 0.018
	4 weeks	0.68 ± 0.30	0.19 ± 0.02	0.96 ± 0.01	520 ± 62	3.9 ± 1.9	0.47 ± 0.05	27 ± 12	0.19 ± 0.07	0.98 ± 0.04	5.2 ± 1.8	0.0078 ± 0.0061

**Table 6:** Elemental composition (at.%) of anodized and non-anodized samples after 3 h and 4 weeks of immersion in the corrosive medium, as determined by EDS analysis.

Time of Immersion	C	O	F	Na	Mg	P	S	Cl	K	Ca	Ti
Before immersion Non-anodized	0.94 ± 1.58	5.2 ± 2.1									94 ± 1
Before immersion Anodized	12.67 ± 3.56	11.92 ± 0.89	2.7 ± 2. 4								72 ± 1
0 week Non-anodized	21.61 ± 6.24	7.6 ± 4.1	1.3 ± 0.7	1.4 ± 1.0						0.20 ± 0.11	67 ± 7
0 week Anodized	17.81 ± 9.16	17.8 ± 3.4	2.0 ± 0.8	0.84 ± 0.45	0.20 ± 0.08	0.67 ± 0.30			0.22 ± 0.12	1.9 ± 1.0	59 ± 1
4 weeks Non-anodized	32.63 ± 10.94	11.5 ± 4.3		2.6 ± 1.3		0.72 ± 0.81	0.18 ± 0.08	1.8 ± 0.8	0.21 ± 0.07	1.0 ± 0.9	50 ± 15
4 weeks Anodized	44.09 ± 11.64	13.7 ± 7.6		2.9 ± 3.0	0.13 ± 0.04	1.2 ± 1.0	0.23 ± 0.06	3.2 ± 3.1	0.39 ± 0.27	1.3 ± 1.0	33 ± 14

## Data Availability

The raw data supporting the conclusions of this article will be made available by the authors upon request.

## Supplementary Materials

The following supporting information can be downloaded at https://www.mdpi.com/article/10.3390/molecules31172959/s1: Figure S1: SEM top view (a) and cross-sectional view (b) of the nanostructured TiO_2_ layer formed on a 3D-printed Ti scaffold. The oxide layer was obtained by anodization at 40 V for 15 min in an ethylene glycol-based electrolyte; Figure S2: Nyquist plots of non-anodized (a) and anodized (b) samples measured in different corrosive media; Figure S3: Nyquist plots recorded after different immersion times in a corrosive media for non-anodized samples (a) and anodized samples (b).

## Abbreviations

The following abbreviations are used in this manuscript:

AM	Additive Manufacturing
3D	Three-dimensional
PVD	Physical Vapor Deposition
CVD	Chemical Vapor Deposition
PBF-LB	Powder Bed Fusion with Laser Beam
OCP	Open-Circuit Potential
FE-SEM	Field Emission Scanning Electron Microscopy
SBF	Simulated Body Fluid
FBS	Fetal Bovine Serum
PBS	Phosphate Buffered Saline
CPE	Constant Phase Element
CP	Commercially Pure
EIS	Electrochemical Impedance Spectroscopy
EDS	Energy Dispersive X-Ray Spectroscopy
