# Supplementary material for: Three-Dimensional Titanium Substrates with Anodic TiO2 Layers for Enhanced Time-Dependent Corrosion Protection in Biomedical Environments
Source: Molecules. 2026 Aug 24;31(17):2959. doi: 10.3390/molecules31172959 (PMC13567555; doi:10.3390/molecules31172959)
Supplement: Supplementary file 1 [file molecules-31-02959-s001.zip › molecules-4400041-supplementary.pdf]

S1.1. Morphology of anodized 3D-printed Ti scaffolds.

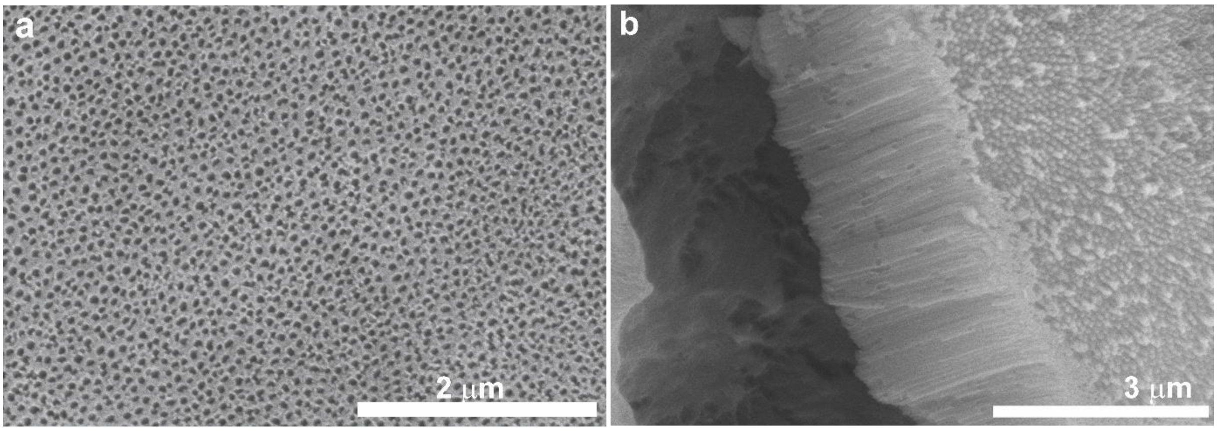

Figure S1. SEM top view (a) and cross-sectional view (b) of the nanostructured TiO_2 layer formed on a 3D-printed Ti scaffold. The oxide layer was obtained by anodization at 40 V for 15 min in an ethylene glycol-based electrolyte.

S1.2. Determination of polarization resistance and corrosion rate from Tafel measurements

Polarization resistance (R_p) was calculated using equation (S1):

$$R_p = \frac{\beta_a \beta_c}{2{,}3 \cdot j_{corr} \cdot (\beta_a + \beta_c)} \quad \text{(S1)}$$

where: j_{corr} is the current density measured at the corrosion potential and β_a, β_c are the anodic and cathodic Tafel constants, respectively.

The corrosion rate was calculated using equation (S2):

$$v_{kor} = \frac{i_{kor} \cdot M}{n \cdot d \cdot A \cdot F} \quad \text{(S2)}$$

where: i_{corr} is the current measured at the corrosion potential M is the molar mass of the material [g mol^{-1}], n is a number of electrons, d is density (4.51 g cm^{-3} [60]), A is the electrode area (9.98 cm^2) and F is a Faraday constant [C mol^{-1}]

S1.3. Impact of corrosion conditions on corrosion resistance

Figure **S2a-b** shows the Nyquist plots recorded for anodized and non-anodized samples in different corrosive media.

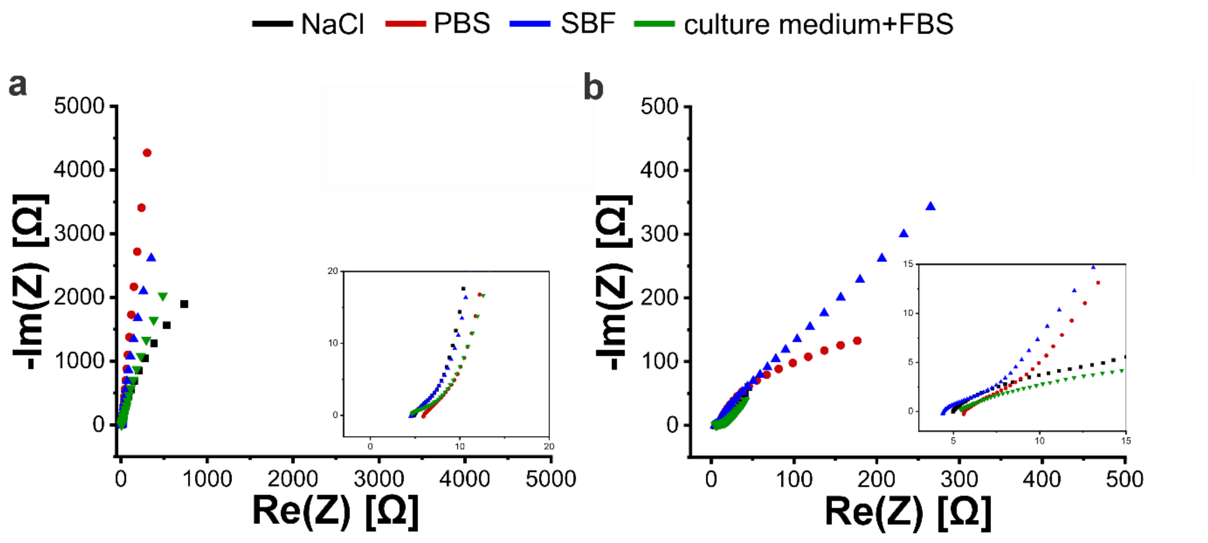

Figure S2. Nyquist plots of non-anodized (a) and anodized (b) samples measured in different corrosive media.

S1.4. Time-dependent corrosion protection

Figure S3 shows a comparison of Nyquist plots for anodized and non-anodized samples after different immersion times in corrosive media.

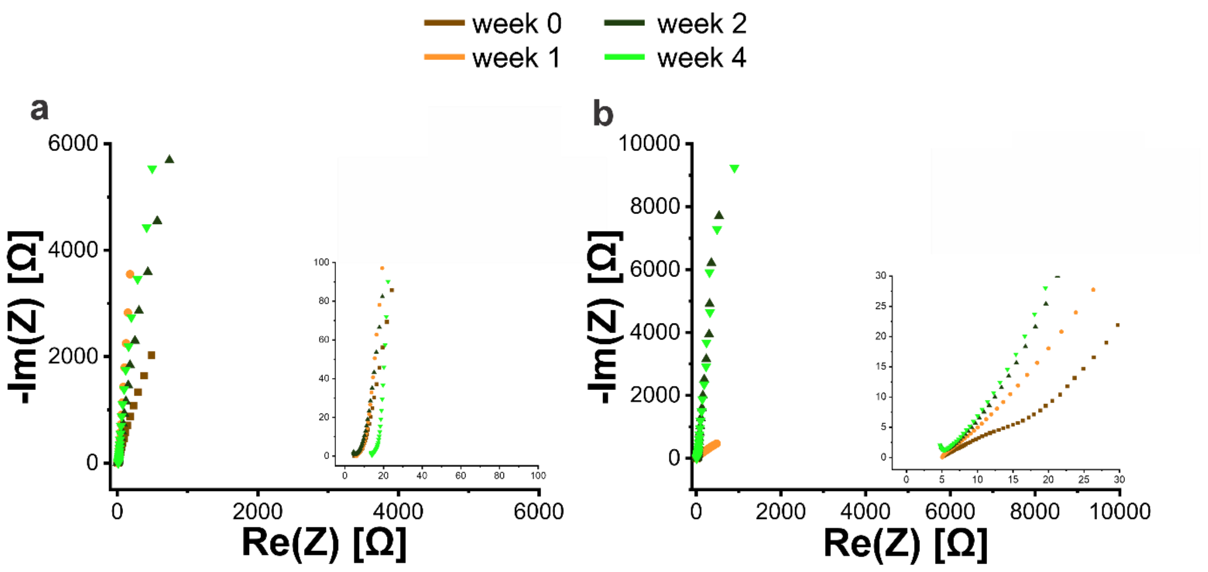

Figure S3. Nyquist plots recorded after different immersion times in a corrosive media for non-anodized samples (a) and anodized samples (b).

The increase in the total surface area resulting from the formation of the nanostructured porous anodic oxide layer for anodized scaffolds was estimated using the equation (S3):

$$A = \pi r^2 + 2\pi r h \quad \text{(S3)}$$

where: r is the pore radius, and h is the pore depth

References

[60] Peters, M.; Hemptenmacher, J.; Kumpfert, J.; Leyen, C. Structure and Properties of Titanium and Titanium Alloys; John Wiley & Sons: Hoboken, NJ, USA, 2003; pp. 1–36. https://doi.org/10.1002/3527602119.ch1.
